# Characteristics of the 100 largest modern zoonotic disease outbreaks

**DOI:** 10.1098/rstb.2020.0535

**Published:** 2021-11-08

**Authors:** Patrick R. Stephens, N. Gottdenker, A. M. Schatz, J. P. Schmidt, John M. Drake

**Affiliations:** ^1^ Odum School of Ecology and Center for the Ecology of Infectious Diseases, University of Georgia, Athens, 30602 GA, USA; ^2^ Department of Pathology, College of Veterinary Medicine, University of Georgia, Athens, 30602 GA, USA

**Keywords:** zoonosis, outbreak, macroecology, transmission mode, virus, bacteria

## Abstract

Zoonotic disease outbreaks are an important threat to human health and numerous drivers have been recognized as contributing to their increasing frequency. Identifying and quantifying relationships between drivers of zoonotic disease outbreaks and outbreak severity is critical to developing targeted zoonotic disease surveillance and outbreak prevention strategies. However, quantitative studies of outbreak drivers on a global scale are lacking. Attributes of countries such as press freedom, surveillance capabilities and latitude also bias global outbreak data. To illustrate these issues, we review the characteristics of the 100 largest outbreaks in a global dataset (*n* = 4463 bacterial and viral zoonotic outbreaks), and compare them with 200 randomly chosen background controls. Large outbreaks tended to have more drivers than background outbreaks and were related to large-scale environmental and demographic factors such as changes in vector abundance, human population density, unusual weather conditions and water contamination. Pathogens of large outbreaks were more likely to be viral and vector-borne than background outbreaks. Overall, our case study shows that the characteristics of large zoonotic outbreaks with thousands to millions of cases differ consistently from those of more typical outbreaks. We also discuss the limitations of our work, hoping to pave the way for more comprehensive future studies.

This article is part of the theme issue ‘Infectious disease macroecology: parasite diversity and dynamics across the globe’.

## 1. Introduction

Disease emergence is widely recognized as a major threat to biodiversity and human health [1–3]. Globalization and land conversion have led to unprecedented mixing of wild species, humans and domesticated animals from previously unconnected biological communities, often causing cross-species pathogen exposure and resulting in the increased emergence of novel pathogens [4,5]. The majority of emerging human diseases, as many as 70% by some estimates [6], are zoonotic, caused by spillover from wildlife and/or via infection of domesticated animals. Because the number of zoonotic outbreaks also appears to be increasing over time [7], gaining a better understanding of the drivers of zoonotic outbreaks is crucial to mitigating disease risks.

**Figure 1.**
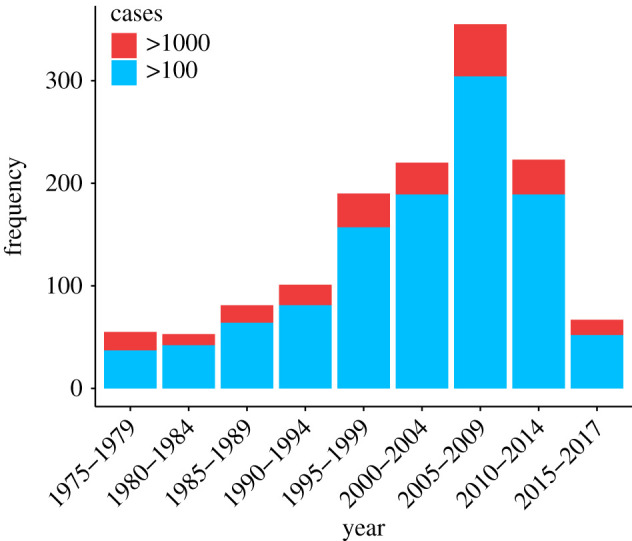
Global numbers of outbreaks with a minimum of 100 cases. Depicts potentially zoonotic outbreaks with at least 100 reported cases and start dates between 1975 and 2017 from Gottdenker *et al*. [11]. Based on these data, it seems most likely that the frequency of large outbreaks is either stable or increasing over time. However, reporting effort, detection capabilities and human population density are all also increasing in many regions over time [12], and a previous study showed that apparent temporal trends in outbreak frequency can vary considerably depending upon the potentially confounding covariates and types of outbreaks (e.g. pathogen taxa) considered [7]. (Online version in colour.)

While disease outbreaks cause considerable distress in aggregate [6–8], it is also true that most outbreaks in modern times are contained relatively quickly. The typical outbreak is limited to fewer than 100 cases [9] and the global impact of most communicable diseases in terms of disability adjusted life years (DALY) lost on an annual basis seems to be decreasing over time [10]. However, large outbreaks that escape control and infect hundreds to thousands of humans or domestic animals still occur regularly (figure 1). For example, an outbreak of salmonellosis in the United States in 1985 infected more than 160 000 people [13] and a 1978 outbreak of the Oropouche virus in Brazil is estimated to have resulted in approximately 227 000 human cases [9]. The second-largest outbreak in recent years was the H1N1 influenza pandemic of 2009–2010, which caused 123 000–395 000 estimated deaths globally [14,15]. Even that pandemic has now been eclipsed by the Covid-19 pandemic, which as of this writing is estimated to have infected 200 million people and caused 4.3 million deaths [16]. Understanding factors that distinguish typical localized outbreaks from large regional epidemics and pandemics is an important challenge in the field of infectious disease macroecology [17]. However, global quantitative studies to date have been limited to overall trends in the number of outbreaks over time [6,7] or patterns of disease diversity [18–20] rather than trends in the factors that cause outbreaks. Here, we discuss the need for quantitative studies of variation in outbreak drivers, as well as some of the challenges in accurately quantifying outbreak dynamics at global scales.

### (a) Exploring the drivers of zoonotic outbreaks

Numerous factors have been implicated as potential drivers of zoonotic outbreaks [11,21,22], including encroachment on wild areas [23–25], biodiversity loss [26,27], climate change [23,28] and socioeconomic factors such as poverty [29,30] and urbanization [31,32]. Many studies have explored the drivers of individual outbreaks and pandemics (e.g. [31,33,34]) or considered risk factors for future spillover or outbreaks of individual diseases [35–37]. For example, several studies have considered how spatial variation in environmental conditions such as temperature and rainfall [36], forest loss [38] and host diversity [39,40] affect overall Ebola virus spillover risk. However, the proportion of Ebola outbreaks in which related factors such as weather conditions, deforestation or human–animal contact played a role as a proximate trigger has not been quantified. In general, no studies of which we are aware have quantified the relative frequency with which these and other environmental and demographic factors contribute to particular outbreaks, or how observed drivers vary with outbreak severity.

There is also a dearth of quantitative work on socioeconomic drivers of zoonotic disease outbreaks. For example, one hypothesis is that international trade and travel contribute to many large outbreaks by providing opportunities for transmission among populations in different countries. Travel was shown to play a role in at least a few large outbreaks such as the 2003 SARS epidemic [41], the H1N1 influenza pandemic [33] and the 2014 Ebola epidemic [42]. However, no studies we know of have quantified the proportion of outbreaks triggered or amplified by international travel, or whether outbreaks in which international travel is important tend to be larger than those confined to a single country. Other socioeconomic factors such as poverty, armed conflicts and variation in public health infrastructure are similar in that they have been investigated for some outbreaks and some diseases [29,43–50] but their overall contribution to disease outbreaks has not been quantified. Even whether the driver profile (i.e. which of multiple potential drivers considered in aggregate contribute to a given outbreak) of large outbreaks tends to differ from that of smaller outbreaks has not been directly tested, nor has the hypothesis that large outbreaks will have more proximate drivers than smaller outbreaks. Few hypotheses about outbreak drivers have been tested quantitatively at global scales.

### (b) Reporting bias and other data challenges

**Figure 2.**
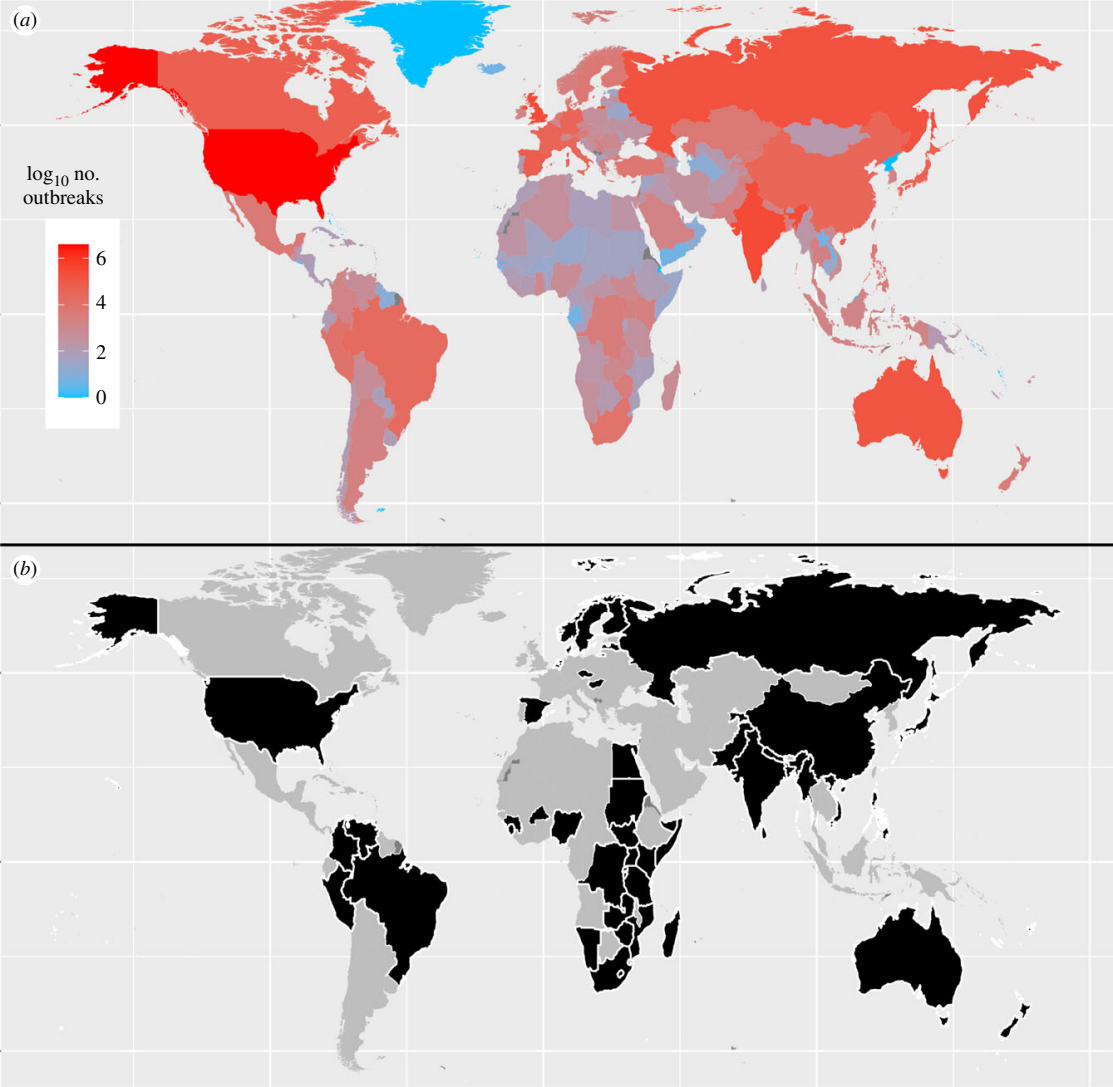
Global distribution of zoonotic outbreaks. Locations of all 4463 potentially zoonotic outbreaks sampled at random for background cases (*a*) and countries that had at least one of the top 100 outbreaks (*b*). Countries in grey lacked outbreaks in each respective dataset.

Complicating global studies of disease trends are attributes of countries, factors that can vary over space and time, that introduce bias to any global dataset of disease or outbreak occurrences [7,51]. Past studies have documented more outbreaks in countries with high gross domestic product (GDP), and in Europe and North America, than lower GDP countries in other regions of the world ([7,23] see also figure 2*a*). It seems unlikely that these countries truly experience more outbreaks than other countries at lower latitudes that are just as populous, and that in many cases have higher overall disease diversity [18,52]. Instead, global outbreak data appear to be biased by factors that vary among countries and regions [7].

One broad class of factors are related to countries’ chances of detecting and reporting outbreaks. For example, it has been shown that countries with larger numbers of Internet users and greater press freedom are more likely to report outbreaks [51,53,54]. Indicators of economic activity such as GDP could be related to variation in health infrastructure and surveillance capabilities, leading to greater chances that outbreaks are detected in more affluent countries [7]. At the same time, poverty is a risk factor for many diseases [55,56], potentially leading to increased risk and greater numbers of outbreaks in impoverished countries. Thus, correlations between GDP and outbreak numbers in either direction could occur. Supporting the hypothesis of detection bias, Smith *et al*. [7] found GDP to be positively correlated with number of known outbreaks per country. Moreover, improvements in values of the human development index (HDI), typically highly correlated with GDP [57], have been associated with reductions in outbreak discovery and communication lag times [58].

Other factors may affect the chance that a country will experience an outbreak, and can also be considered drivers themselves in some contexts (see below). Ultimately, the availability of hosts is perhaps the largest single risk factor for outbreaks [5]. Population density might, therefore, be expected to be the most important aspect of demographic variation due to its influence on transmission rates [59]. However, Smith *et al*. [7] showed that total human population size was more strongly correlated with number of outbreaks (across countries) than population density, regardless of the subset of outbreaks considered. Latitude has also been shown to be related to disease diversity, with tropical countries showing greater diversity [18,52] than high latitude countries. This could reflect the influence of environmental conditions that vary with latitude (e.g. [60,61]). Lower latitude assemblages also contain higher mammalian and avian host diversity [62,63], which has been shown to be positively correlated with outbreak and disease emergence risk [23,64].

Whether factors affecting disease diversity and outbreak risk should be considered a source of bias will vary somewhat with the question of interest. For instance, in a study of the effects of an anthropogenic driver such domestic livestock production on outbreak risk, latitude would be regarded as a confounding factor. However, if the goal of a study is to generate an accurate statistical model of spatial variation in disease diversity, latitude would be regarded as an important predictor variable.

Smith *et al*. [7] was among the first quantitative global-scale outbreak studies to control for variation in detection capabilities, reporting effort and disease diversity among countries. They showed that global outbreak frequency consistently increased over time in analyses of raw data. However, this trend was often diminished or absent in models that included covariates such as latitude, GDP and population density. What effect, if any, these factors would have on statistical models of variation in outbreak drivers has not been explored. However, it might be expected that at least reporting bias (e.g. press freedom) might influence such analyses.

### (c) Case study: drivers of the 100 largest bacterial and viral zoonotic outbreaks in recent history

To illustrate these issues, we studied the variation in the frequency of drivers reported in a sample of cases from a global dataset of 4463 outbreaks of bacterial and viral zoonotic pathogens. We describe these data in more detail below. They were derived from the GIDEON Guide to Outbreaks [9], which collects information from the same sources as two previous global studies of outbreak diversity and frequency [6,7]. Here, we focus on whether the driver profiles of the largest 100 outbreaks in the dataset, in terms of case numbers, are different from those of 200 randomly chosen controls. In statistical terms, we compare the tail of the distribution to a random sample.

We scored outbreaks using criteria reflecting drivers discussed in published reviews (e.g. [21,22]). Our approach was designed to represent a variety of different kinds of drivers including ecological [24,27,53], environmental [23,28] and socioeconomic [29,30,65] factors. In total, we evaluated the potential influence of 48 different drivers on each outbreak (electronic supplementary material, table S1). We also consider whether apparent differences in the frequency with which each of these factors is important in large versus background outbreaks is robust when analyses include variables reflecting variation in reporting and disease diversity among countries and over time (following [7]). Finally, we consider broad differences in the characteristics of pathogens, including whether viral or bacterial pathogens more frequently cause large outbreaks, and testing for the influence of transmission mode (e.g. direct versus environmental transmission).

## 2. Case study materials and methods

### (a) Sampling and scoring outbreaks

We identified candidate outbreaks of zoonotic pathogens from the GIDEON Guide to Outbreaks [9] based on the diseases reported. GIDEON defines an outbreak as a number of clustered cases which is higher than the average or expected incidence for a region where the cases occur. Functionally it also tends to be limited to events recognized and reported by health agencies (all outbreaks that we scored) and events of less than 2 years duration (96.5% of outbreaks in our full dataset of 4463 outbreaks). See online supplementary material (electronic supplementary material, S2 Additional Methods) for additional details of outbreak sampling procedures.

Those outbreaks we considered potentially zoonotic were caused by pathogens that can be transmitted between animals and humans (e.g. West Nile virus, hantavirus, Q fever), though individual outbreaks included were often not of zoonotic origin (e.g. most outbreaks of hepatitis E). We excluded opportunistic pathogens (e.g. *Pneumocystis carinii*, *Aspergillus* sp.), but did include some diseases caused by both zoonotic and non-zoonotic pathogens (e.g. tuberculosis). We focused on viruses and bacteria because they are the broad taxa that cause outbreaks most frequently (e.g. fewer than 10% of outbreaks we considered including were caused by eukaryotic parasites). We discuss the rationale for our criteria (electronic supplementary material, table S2), including the inclusion of ‘borderline’ diseases such as tuberculosis and those of some arboviruses, in the online supplementary materials (electronic supplementary materials, S2.1 Diseases included and excluded). In general, we included diseases classified as zoonotic by working groups of the CDC [66], the UK Health Ministry [67] and the Pan American Health Organization [68].

In preliminary analysis, we found that many of the largest outbreaks were from sparsely sampled time periods. For instance, in the full dataset all but one of the ten outbreaks from before 1800 were among the 100 largest, and more than half of the largest outbreaks occurred in poorly documented years (less than five recorded outbreaks) before the invention of antibiotics. To understand contemporary outbreaks, we focused on well-characterized years with 20 or more documented outbreaks per year from 1974 to the present. Five of the seven covariates that we used to characterize potential sample bias (see below) could also be quantified throughout this time interval. With this cut-off, we produced a final dataset of 4463 contemporary outbreaks caused by zoonotic pathogens, within which we compared the putative drivers of the 100 largest (defined by minimum estimated number of cases) to those of 200 random background or control outbreaks (electronic supplementary material, figure S1).

To score outbreaks, we compiled a list of 48 potential drivers based on factors discussed in reviews of zoonotic outbreak literature [11,21,22,69]. Drivers were chosen to represent a variety of phenomena including ecological, environmental and socioeconomic factors (electronic supplementary material, table S1). For each outbreak, drivers mentioned in sources such as peer-reviewed publications cited in GIDEON [9], Morbidity and Mortality Weekly Reports [70] and ProMed emails [71] were noted. Each of the 48 drivers was then scored as either not reported to contribute to an outbreak (0) or reported as a likely contributing factor by at least one source (1). We wished to quantify the frequency with which human–animal contact and other factors appear to be proximate drivers of large versus typical outbreaks of zoonotic diseases. Because any outbreak in which human–animal contact did not appear to be a factor would not be considered zoonotic in the strictest sense, we refer to our data as ‘potentially zoonotic’ outbreaks.

### (b) Statistical analyses

We conducted all analyses in R v. 4.0.0 [72]. We first ran contingency table analysis, a permutation test of independence implemented in the R package coin [73], to determine whether the overall frequency of reported drivers differed between large outbreaks and controls. Analyses excluded drivers reported in less than 3% of outbreaks (i.e. found in fewer than nine outbreaks); we observed no significant (*α* = 0.05) differences in the frequency of such drivers between large and background outbreaks. We repeated this analysis using drivers found in at least 5% of outbreaks (15 or more outbreaks), and then on the three drivers that differed the most between large and background outbreaks.

We then conducted *χ*^2^ analyses of each individual driver to determine when the frequency of a driver being associated with an outbreak differed in top 100 versus background outbreaks. We report the results of both multivariate analyses testing differences in the overall driver profile of large and background outbreaks, and univariate analyses that maximize statistical power by focusing on individual drivers. In the latter analyses, we highlight results still significant at *α* = 0.05 after applying a Bonferroni correction for 48 simultaneous comparisons (only *p*-values < 0.001 are considered significant).

Finally, we investigated the potential impact of factors reflecting differences in reporting effort, detection capabilities and disease numbers (i.e. disease diversity and perhaps prevalence or transmission rates) among countries. We refer to these collectively as ‘sample bias covariates’ since we are primarily interested in whether differences in the reported drivers and pathogens of large versus background outbreaks are statistically significant after accounting for their influence. We do not mean to imply that variables such as human population density lack any functional relationship with outbreak size. Following a previous global study of disease outbreak patterns [7], we used GDP, press freedom, Internet use, population size, population density and latitude as covariates to control for sample bias. We also included the number of phone subscriptions per hundred individuals, as data were available for the entire time range our dataset covered and we would expect it to have a similar effect on reporting to Internet use. Whenever possible, each of these seven covariates was quantified for the year and country in which an outbreak in our dataset was reported. Data for most covariates come from the World Bank [12]. Latitude was based on the latitudinal centroid of each country included in our analyses [74]. (See electronic supplementary material, dataset S3 for a full description of these data.)

Logistic regression models were run with and without sample bias covariates. Due to differences in the time ranges of covariates, and in the number of countries and years for which data were available even within the time ranges covered, sample sizes varied considerably. In models with 300 or fewer observations, we were also concerned that including too many predictor variables might inflate rates of type II error [75]. To ensure that our qualitative results were not unduly influenced by these factors, we included results from a wide variety of logistic regression models including (i) models with no sample bias covariates, (ii) models with all covariates, (iii) models only including covariates measured over the entire time range of outbreaks in our study and (iv) models considering each covariate individually. This led to a total of more than 370 models (see electronic supplementary material, supplemental table appendix). For the sake of brevity, we only report coefficients of the relationship between the predictor of interest (either a driver, pathogen type or transmission mode) and the response variable.

### (c) Pathogen characteristics

We also quantified variation in the biological and transmission characteristics of diseases based on the identity of their causative pathogens, or range of pathogens for diseases that can be caused by multiple species. From standard veterinary and medical references [76–79], we determined whether pathogens were viral or bacterial and their (non-exclusive) modes of transmission (see electronic supplementary material, dataset S1 for a full reference list). Definitions used to score the transmission modes of pathogens followed Antonovics *et al*. [80]. Vector-borne pathogens were those that sometimes infect hosts through contact with an arthropod vector such as a mosquito or tick. Directly transmitted pathogens were those that can be transmitted by close contact between hosts, including but not limited to direct ecological interactions (e.g. predation) and sexual transmission. Environmentally transmitted pathogens were those that can be transmitted through contaminated soil or water, airborne pathogens and/or fomites. We made no attempt to distinguish which mode of transmission was most prevalent in any particular outbreak. We used *χ*^2^ analyses and multivariate logistic regression models, including and excluding sample bias covariates, to test for differences in the characteristics of pathogens causing large outbreaks versus controls.

## 3. Case study results and discussion

### (a) Outbreak drivers

**Figure 3.**
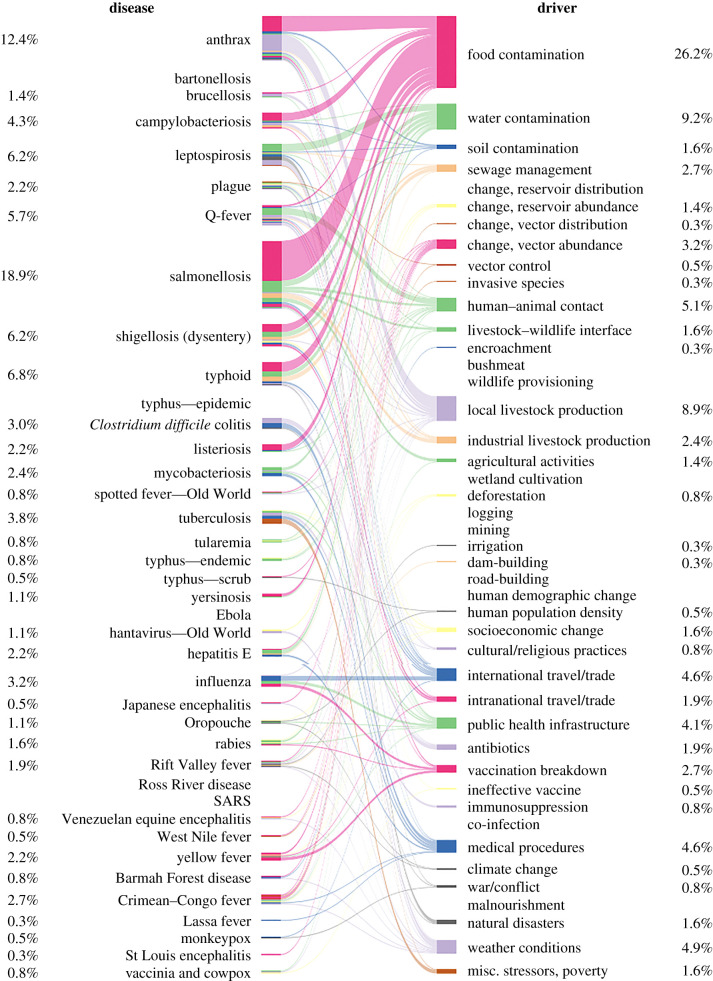
Frequency of diseases and outbreak drivers in a sample of 200 ‘background’ outbreaks. Bipartite network relating diseases to causal drivers of the background outbreaks. Percentages and widths indicate the relative number of times each driver or disease was scored across outbreaks. Colours are purely for illustrative purposes, to help visualize the relative contribution of different drivers to different diseases.

**Figure 4.**
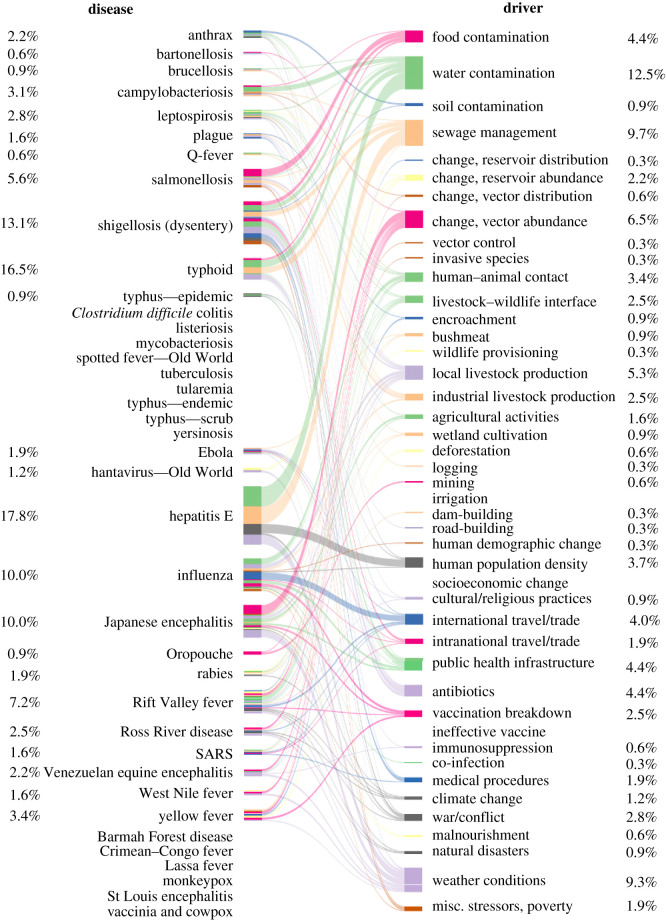
Frequency of diseases and drivers in the 100 largest zoonotic outbreaks since 1974. Bipartite network relating diseases to causal drivers of the 100 largest outbreaks. Percentages and widths indicate the relative number of times each driver or disease was scored across outbreaks. Colours are purely for illustrative purposes, to help visualize the relative contribution of different drivers to different diseases.

The driver profile of large outbreaks differed from that of background outbreaks, regardless of the definition of the background used (tables 1 and 2, figures 3 and 4; electronic supplementary material, tables S3 and S4). More proximate drivers were associated with larger outbreaks than controls (electronic supplementary material, table S11). The mean number of drivers was 3.19 for large outbreaks and 1.91 for controls (random background outbreaks)—perhaps reflecting a tendency for large outbreaks to be precipitated by interactions (e.g. feedbacks) among multiple drivers.

**Table 1.** Contingency table analysis of drivers scored in the top 100 versus random background outbreaks. We considered 48 potential drivers, but many of them were rarely observed in the outbreaks we scored. Cut-off lists the percentage of outbreaks that a driver needed to be scored in to be included in a given contingency table analysis. Analyses are presented including and excluding covariates that have been found to confound patterns of disease occurrence and reporting in past studies. ‘All covariates’ indicates analysis including drivers scored for each outbreak as well as the following variables for the country and year in which an outbreak was reported: per capita GDP, Internet users per 100 individuals, phone lines per 100 individuals, press freedom, human population density, human population (total) and latitude, whereas ‘1974 covariates’ indicates analyses in which Internet use and press freedom (which were only measured after 1990) are excluded. ‘*N*’ indicates the number of rows of complete case data (see Methods for additional details).

cut-off	predictors	*N*	χ^2^	*p*-value
3% or more (no covariates)	20	300	101.250	<0.0001
3% or more (1974 covariates)	20 + 5	290	47.075	<0.0001
3% or more (all covariates)	20 + 7	160	21.155	0.0017
5% or more (no covariates)	15	300	92.012	<0.0001
5% or more (1974 covariates)	15 + 5	290	47.076	<0.0001
5% or more (all covariates)	15 + 7	160	21.156	0.0017
top three (no covariates)	3	300	52.371	<0.0001
top three (1974 covariates)	3 + 5	290	47.069	<0.0001
top three (all covariates)	3 + 7	160	21.155	0.0017

**Table 2.** Drivers that differed between large and random background outbreaks with a *p*-value < 0.1. The *p*-values for all other drivers were > 0.1. Rows in italics indicate *p* < 0.05, rows in bold are still significant after applying Bonferroni correction for 48 independent comparisons (i.e. *p* < 0.001). Results for variables not bolded often differed when models included covariates accounting for the attributes of countries where outbreaks occurred (see electronic supplementary material, tables S13–S39).

driver	±	top 100%	background%	χ^2^	*p*-value
change in reservoir abundance	+	7	2	3.409	0.0648
*war/conflict*	*+*	*9*	*2.5*	*4*.*954*	*0*.*0260*
*human population density*	*+*	*11*	*3*	*6*.*555*	*0*.*0105*
*antibiotics*	*+*	*14*	*4*	*8*.*394*	*0*.*0038*
**water contamination**	**+**	**40**	**20**	**12**.**633**	**0**.**0004**
**sewage management**	**+**	**31**	**10**	**19**.**375**	**<0**.**0001**
**change in vector abundance**	**+**	**21**	**3.5**	**22**.**103**	**<0**.**0001**
**weather conditions**	**+**	**29**	**6.5**	**26**.**194**	**<0**.**0001**
**food contamination**	−	**14**	**48**	**31**.**739**	**<0**.**0001**

Another notable result is that many drivers we considered were implicated in very few outbreaks (table 3; electronic supplementary material, tables S1 and S5). For example, urbanization, logging and road building were discussed as possible outbreak drivers in Gottdenker *et al*. [11], but were found in at most one outbreak out of 300. Moreover, at least 20 drivers (table 3; electronic supplementary material, table S5) played a role in less than or equal to 1% of outbreaks. However, each driver included in our list (electronic supplementary material, table S1) has been discussed in reviews of the factors driving modern outbreaks and/or emerging infectious diseases (e.g. [11,22]) and is likely important in some systems. For example, bushmeat consumption, capture and processing was only implicated as a driver in four outbreaks in our study, yet was associated with disease spillover events that caused several Ebola outbreaks [81–83], including at least one cluster of cases with more than 200 fatalities [84]. In a follow-up study underway, we found bushmeat contributed to nearly 50% of Ebola outbreaks [85]. Some drivers may have had low frequency in our data due to systematic biases in the literature we used to score outbreaks. Key sources (e.g. ProMed emails [71] and Morbidity and Mortality Weekly Reports [70]) are primarily written by clinicians rather than ecologists or sociologists. Changes in reservoir abundance or demographic changes in human populations may be less often considered by clinicians than specialists in other disciplines.

**Table 3.** Drivers rarely reported in outbreaks we scored. Number and percentage refer to the random background outbreak data (*N* = 300). Note that drivers rare in our sample could nevertheless be highly influential in some disease systems (see text for example).

driver	number	percentage
aquaculture	0	0.00
irrigation	0	0.00
reforestation	0	0.00
urbanization	0	0.00
dam building	1	0.33
famine^a^	1	0.33
human demographic change	1	0.33
ineffective vaccine	1	0.33
introduced/invasive species	1	0.33
logging	1	0.33
road building	1	0.33
wildlife provisioning	1	0.33
change in reservoir distribution	2	0.67
co-infection	2	0.67
mining	2	0.67
change in vector control	3	1.00
immunosuppression	3	1.00
malnourishment	3	1.00
wetland cultivation	3	1.00

^a^Preliminary analyses showed that famine was a reported driver of several large outbreaks prior to 1974.

Perhaps the most consistent qualitative characteristic of the drivers of the largest outbreaks, such as weather conditions and contamination of water supplies, was that they operated over large scales (table 2; electronic supplementary material, table S4), though we lacked a rigorous way to group drivers into ‘broad-scale’ or ‘narrow-scale’ *a priori*. Furthermore, even a driver that often operates at small scales such as food contamination [86] can affect a wide geographical area under the right conditions [13], and many factors that presumably generally operate at large spatial scales such as changes in the geographical distribution of reservoirs and urbanization were rarely reported as contributing to outbreaks (table 3). Future studies could quantify the typical spatial or temporal extent of different classes of outbreak drivers to test directly for a correlation with the case numbers or the size of regions affected.

Whether model results for a given driver were statistically significant varied considerably depending upon the covariates included and whether we used a truly random background or only included outbreaks with typical case numbers (electronic supplementary material, tables S13–S39). However, results for four drivers were extremely robust across model specifications (electronic supplementary material, tables S15, S16, S25, S26, S28, S29, S38, S39), and always statistically significant. Unusual weather patterns, changes in vector abundance and water contamination, usually representing contamination of water supplies, were much more commonly found in large outbreaks than in controls (table 2; electronic supplementary material, tables S4, S15, S25, S26, S28, S38, S39). The importance of these eco-environmental drivers is especially surprising given the potential bias in our data sources towards clinical drivers. The importance of changes in vector abundance is also somewhat surprising given that we excluded malaria from our analyses. Frequently in large outbreaks, it was reported that a month of unusually high rainfall caused a population explosion of vectors such as mosquitoes, which led, in turn, to many cases of vector-borne illness [87–89]. In fact, both weather conditions and changes in vector abundance were putative drivers of 16 of the 100 largest outbreaks. Poor sewage management (including sewage system failures), in part a socioeconomic factor, was also frequently recognized as a driver of large outbreaks (table 2; electronic supplementary material, tables S4, S23, S36). Sewage management could also be considered an environmental hazard, as it was a contributing factor in roughly half of the cases involving water contamination (electronic supplementary material, tables S8 and S9).

Our results also imply that failures of societal and medical resources may tend to be important in large outbreaks. War or large-scale conflict was four times as likely to be found among drivers of large compared to background outbreaks (table 2; electronic supplementary material, table S4). Unsurprisingly, large outbreaks were more likely to start in areas of unusually high human population density, possibly straining social and medical resources. Antibiotic resistance was also three times as frequent in large outbreaks, perhaps rendering normal medical interventions ineffective. While intriguing, patterns for war, population density and antibiotic resistance were not as strongly supported (*p* > 0.001; table 2), and were often not statistically significant (*α* = 0.05) in analyses including sample bias covariates (electronic supplementary material, tables S13–S39).

### (b) Pathogen characteristics

One might expect only a few common zoonotic pathogens with high transmission rates such as salmonella [90], influenza [91] and typhoid [92] to have the potential to cause large outbreaks with thousands of cases. From this perspective, the diversity of diseases (*n* = 27) across the 100 largest outbreaks was surprising. In a sample completely random with respect to case numbers and twice as large we only observed 33% more diseases (i.e. a sample of 200 outbreaks from a global dataset included 35 diseases). This suggests that specific pathogens with the potential to cause large outbreaks will be hard to anticipate, though they did have a tendency to be viral and use vector-borne transmission more frequently than the diseases of background outbreaks (table 4; electronic supplementary material, tables S10, S41, S42, S45, S46). However, the relative frequency of diseases was somewhat different between the two datasets (electronic supplementary material, table S5). Typhoid (including enteric fever) and shigellosis (dysentery) were among the five most common diseases in both the background and top 100 outbreaks (electronic supplementary material, figure S2). By contrast, the rest of the top five differed considerably, with three bacterial diseases (salmonellosis, anthrax and tuberculosis) and three viral diseases (hepatitis E, influenza and Japanese encephalitis) rounding out the background and top 100, respectively. Perhaps related to the high frequency of anthrax and salmonellosis, food contamination was much more commonly found to be a driver in the background than in large outbreaks (table 2; electronic supplementary material, tables S3, S16, S29).

**Table 4.** Pathogen characteristics of large versus random background outbreaks. Type indicates whether the pathogen causing an outbreak was viral or bacterial. Transmission indicates the transmission modes of a pathogen. The latter categories are not exclusive, some pathogens are transmitted by all three modes. This table is primarily meant to summarize qualitative patterns of variation. Results for transmission modes varied considerably when analysed using variables accounting for variation in the attributes of countries where outbreaks occurred (see electronic supplementary material, tables S40–S47).

type	top 100%	background %	χ^2^	*p*-value
virus	58	17.5	49.245	<0.0001
transmission	top 100%	background %	χ^2^	*p*-value
vector	27	11	11.345	0.0008
direct	55	72.5	8.417	0.0037
environmental	73	84.5	4.940	0.0269

The overall biological profile (taxon and transmission modes) of pathogens that cause large outbreaks was also different from that of controls (table 4; electronic supplementary material, table S10). Large outbreaks were more likely to be caused by viral than bacterial pathogens (table 4; electronic supplementary material, figure S3 and tables S10, S40, S44), possibly because widespread use of antibiotics in modern times has often been effective in preventing large bacterial outbreaks. Two lines of evidence support this interpretation. First, antibiotic resistance was a more frequent driver of large outbreaks than controls (table 2; electronic supplementary material, tables S4, S13, S27). Second, in preliminary analyses including outbreaks from before the invention of antibiotics, bacterial pathogens were much more common in large outbreaks. Among the 18 large outbreaks from before 1930 (treating the 1918–1919 worldwide flu pandemic as a single outbreak), 14 were caused by bacterial pathogens.

Results related to transmission mode were less clear. Vector-borne transmission was overall the least common transmission mode, but was much more common among pathogens of large outbreaks versus controls (table 4) despite the exclusion of malaria from our study. The pathogens causing large outbreaks also relied on direct and environmental transmission less frequently than those found in background outbreaks (table 4), but environmental transmission was still the most common transmission mode used by pathogens in both sets of outbreaks. However, directly transmitted pathogens caused greater than 50% of both large and background outbreaks. None of the differences in the frequency of transmission modes were significant in models including variation in per capita numbers of phone lines (electronic supplementary material, tables S41–43, S45–47), suggesting reporting bias affected the outcome of these analyses. One possibility is that key diseases (e.g. vector-borne diseases) get reported less frequently in countries with poor communication infrastructure, generating an apparent relationship in analyses that do not take it into account (table 4).

### (c) Case study conclusions

Overall, our findings show that the profile of a large outbreak that escapes control and includes thousands of cases differs considerably from that of a more typical outbreak. Water contamination was the most common driver of large outbreaks (median number of cases: 7933.5), followed by poor sewage management, unusual weather conditions and changes in vector abundance (figure 4, table 2; electronic supplementary material, table S4). Pathogens that caused large outbreaks tended to be viral, were more likely vector-borne, and less likely to be transmitted directly or environmentally (table 4; electronic supplementary material, table S10). Among background cases (median number of cases in background outbreaks: 42.5), food contamination was the most common driver, followed by water contamination, local livestock production and human–animal contact (figure 3). Pathogens causing these outbreaks tended to be bacterial and were considerably less likely to be vector-borne (table 4; electronic supplementary material, table S10, figure S3). Importantly, these results were not driven by the higher frequency of outbreaks caused by *Salmonella* in controls versus large outbreaks (electronic supplementary material, tables S6 and S7).

## 4. Implications for future work

### (a) Sample bias and quantifying outbreak drivers

We show that important insights can be gained by applying a simple driver schema (electronic supplementary material, table S1) to global outbreak data. However, one of our primary results, that large outbreaks tend to have more proximate drivers than background outbreaks (electronic supplementary material, table S11), could at least be partially driven by investigator bias. It is expected that more research attention will focus on large outbreaks, particularly those with many fatalities, great economic consequences, or other dramatic effects. It is thus possible that the factors driving large outbreaks tend to be more fully documented than those of background outbreaks, which tend to be smaller even when chosen fully at random (electronic supplementary material, figure S1). Undoubtedly, in at least some cases, the factors influencing outbreaks are straightforward, and some outbreaks would have fewer drivers noted regardless of the study effort applied to them. For example, an outbreak of food poisoning with less than a dozen cases traced to one batch of food in a particular household [93] almost certainly has fewer proximate drivers than the 2009–2010 worldwide flu pandemic [15]. We also confirmed that neither differences in numbers of drivers (electronic supplementary material, table S11) nor all differences in the frequency of drivers (electronic supplementary material, tables S13–S40) resulted from patterns of expected disease frequency or reporting bias [7] across years and countries.

However, results for some drivers, such as armed conflicts (electronic supplementary material, tables S24 and S37), changes in reservoir abundance (electronic supplementary material, table S14) and industrial livestock production (electronic supplementary material, tables S18 and S31) often varied depending on the covariates considered. Thus, between-country differences in resources (e.g. GDP), human demographics (e.g. total population and population density), communication infrastructure (e.g. phone lines and Internet users in our study) and expected disease diversity (e.g. latitude) are important to control for in any global analysis of outbreak characteristics. For the most part, we considered the same covariates included in a previous study of global trends in outbreak frequency [7]. However, we also included per capita numbers of phone lines [12], and this proved to vary more strongly between large and background outbreaks than any other covariate we considered (electronic supplementary material, table S11). We speculate that the negative correlation we observed between the chances that an outbreak was among the top 100 and numbers of phone lines (electronic supplementary material, table S11) reflects a tendency for smaller outbreaks to be detected and reported more frequently in countries with robust communication infrastructure, and thus a bias in the distribution of background cases (figure 2*a*). Regardless, many of our results proved to be robust to the effects of this and other sample bias measures (e.g. table 1; electronic supplementary material, tables S11, S15, S16, S23, S25, S26, S28, S29, S36, S38, S39).

The number of years and countries for which each covariate could be quantified varied greatly. Our measure of press freedom has only been tracked since 2001 [94] and Internet use was effectively zero in most countries before the very late 1980s [95]. Even for well-sampled variables, data for a few (less than three) rows were often unavailable and did not match up between covariates, further reducing sample sizes in complete case models. Therefore, to maximize the statistical power of models, we ran models with different combinations of better-sampled covariates (e.g. electronic supplementary material, tables S13–S47). For this case study, we relied on statistical methods (e.g. logistic regression and *χ*^2^) that we assumed would be familiar to most readers. Future studies could employ boosted regression trees [96] or other methods that do not require complete case analysis, or could impute missing data values [97].

Number of physicians, hospitals, or health spending as a percentage of GDP might more directly reflect healthcare resources and disease reporting. However, data on these variables were too limited temporally and geographically to be useful for global analyses, at least from current publicly available sources [12]. Future large-scale studies might focus on areas such as Europe and North America where data for these variables are more often available. Another alternative approach would be to focus on patterns in individual countries in which many outbreaks have been reported. For example, in the global dataset that we sampled there were 786 rows from the US and 292 rows from India.

### (b) Different study systems and related questions

We considered only potentially zoonotic outbreaks of viral and bacterial pathogens. Outbreaks of protozoal diseases such as malaria and of human pathogens such as HIV/AIDs are at least as much of a health concern as the diseases we consider here [10], and more quantitative studies of the factors that commonly drive them are badly needed. One of the reasons that we focused on zoonotic pathogens is that a greater range of drivers are likely potentially relevant to them. For example, we expect that human–animal contact, deforestation and bushmeat consumption play little role in pathogens maintained almost entirely by human-to-human transmission such as dengue [98] or sexually transmitted disease such as syphilis [99]. However, this is not to imply that outbreaks of such diseases [100–102] are of less interest.

We derived a dataset of outbreaks from the information included in GIDEON [9]. However, the data that we could extract directly from this source was often limited, and the additional information that was available in reviews or compilations focused on specific pathogens (e.g. [103,104]) varied widely. We found we could always assign an outbreak to a country and a range of years. However, of 8431 outbreaks that we originally considered (see electronic supplementary material, table S2 for pathogens included), number of cases was only available for 4930 (fewer when limited to those after 1973), and deaths were only reported for 1534. In most outbreaks, no information on drivers was available. We thus had to investigate the reported drivers of each outbreak by intensive searches of primary literature. This was one reason why we chose to compare the tail of the outbreak distribution to a random sample of the rest. It allowed us to address a question we thought would be of considerable interest while only scoring a few hundred outbreaks (i.e. the hundred largest and a comparable sample of the background).

Our overarching study goal was to characterize the driver and pathogen profiles of the largest zoonotic outbreaks in recent history. However, we used a case-control framework to do this, an approach with some limitations [105,106]. Another way to investigate variation in outbreak severity would be to consider outbreak size or mortality as a continuous variable. The factors associated with the largest outbreaks could be similar to or distinct from those driving differences in the number of cases or deaths. Future studies could build statistical models to better understand the overall variation in outbreak size. Studies have attempted to predict the size of outbreaks of individual diseases based on properties of human pathogen networks, initial host population sizes or pathogen transmissibility [107–109]. However, no studies of which we are aware have included variation in outbreak drivers in models or looked at realized outbreak sizes across large numbers of diseases to test for general relationships. We consider this an important but distinct question from the one we focused on.

We also believe that building accurate statistical models of outbreak severity as a continuous response variable would require driver data for many more outbreaks than we present here. Given the transmission characteristics of different pathogens [80], the factors that tend to drive outbreaks of any given disease likely vary considerably. For example, in a comprehensive study of filovirus outbreaks currently underway [85], we found that socioeconomic factors such as poverty and degraded health infrastructure are much more important in filovirus outbreaks [103] than outbreaks included in the current study. Similarly, the factors contributing to variation in outbreak size likely vary among other diseases. To accurately characterize patterns of variation in case numbers, it would likely be necessary to build statistical models of the driver profile of outbreaks within versus across diseases and regions. This might require data on the profiles of thousands of outbreaks for a truly global analysis including many diseases. Other response variables such as mortality [110,111] or economic impact [111,112] might also be of more interest than the case number for many questions.

The dataset from which we sampled outbreaks would likely be sufficient for a global analysis of case numbers if the drivers of every outbreak (*n* = 4463 with reported numbers) were scored. However, scoring so many records using the methods we employed would have required us to review tens of thousands of primary references. Machine learning methods such as natural language processing (NLP) [113] and neural joint models [114] might be used to help automate this process. Data similar to what we present might be useful for parameterizing models based on the text passages used to score drivers of outbreaks. Though NLP is not yet widely used in macroecology, it has been successfully used to build databases of host–parasite association in previous studies [115,116]. Broader use of NLP and related machine learning methods (e.g. [117,118]) to generate more detailed and complete databases of outbreak characteristics represents an exciting avenue for future work. The key to leveraging such data effectively will be more collaborative work where statistical models are co-produced by experts in environmental and socioeconomic drivers, stakeholder issues and policy (e.g. [119,120]).

## 5. Conclusion

In a future in which large zoonotic disease outbreaks will almost certainly continue to occur regularly (figure 1), a better general understanding of the factors affecting variation in the severity of outbreaks is critical to the wellbeing of the global community. Here, we present proof-of-concept work comparing the drivers of the largest outbreaks in a global dataset of zoonotic bacterial and viral pathogen outbreaks to similar background outbreaks. We find the driver and pathogen profile of the largest outbreaks varies considerably from two sets of generally smaller (in terms of case numbers) random background outbreaks, a result that proved extremely robust. We discuss many of the challenges inherent in macroecological studies of outbreak dynamics. Data on disease or outbreak occurrences that spans the globe will undoubtedly be somewhat biased by large differences in reporting effort and detection capabilities among countries, and over time. We suggest that a promising way forward will be via more comprehensive studies that consider number of cases or other outcomes (e.g. mortality, duration, region affected, economic impact) as continuous variables.

## Data accessibility

All statistical analyses were conducted using base functions in R and the coin library. Data used for analyses are included in this submission as electronic supplementary material, datasets S1–S4.
